# Validation of Low-Cost Impedance Analyzer via Nitrate Detection

**DOI:** 10.3390/s21196695

**Published:** 2021-10-08

**Authors:** Dirk Johannes De Beer, Trudi-Heleen Joubert

**Affiliations:** Carl and Emily Fuchs Institute for Microelectronics (CEFIM), University of Pretoria, Pretoria 0002, South Africa; trudi.joubert@up.ac.za

**Keywords:** impedance spectroscopy, low-cost, water quality monitoring, nitrate detection, point-of-need

## Abstract

Impedance spectroscopy is a widely used electrochemical technique with a wide variety of applications. Many of these applications benefit from the additional accessibility provided by low-cost impedance devices. With this in mind, a low-cost impedance device was designed for a high performance-to-cost ratio. The performance of this analyzer was validated against a high-performance DropSens µStat-i 400s potentiostat by performing an application-based experiment. Nitrate detection provides a relevant experiment because of the importance of maintaining precise nitrate concentrations to mitigate the impact of nitrate fluctuations on the environment. Dissolved nitrate samples of different concentrations, in the range 3–1000 mg/L, were confirmed colorimetrically and measured with both instruments. A calibration curve of the real impedance matched a sigmoidal transfer, with a linear region for concentrations below 10 mg/L. The device under investigation exhibited an average magnitude error of 1.28% and an average phase error of 0.96${}^{\circ }$ relative to the high-performance standard, which validates the performance of the low-cost device. A cost analysis is presented that highlights some of the complexities of cost comparisons.

## 1. Introduction

### 1.1. Impedance Spectroscopy

Electrochemical impedance spectroscopy (EIS), or simply impedance spectroscopy, is an electrochemical technique used to measure the impedance of a system [1]. This is typically achieved by varying the frequency of an applied voltage or current waveform across an electrode and measuring the resultant current or voltage to determine the impedance. The impedance, *Z*, of a system is defined as

(1) $$Z=\frac{V}{I}$$

where *V* and *I* are the complex voltage and current across the measured system. The impedance is therefore also a complex quantity with both a magnitude and a phase (in a polar coordinate system). Practically, the magnitude, |Z|, and phase, ∠Z, of the impedance can be determined separately in a system according to

(2) $$\left|Z\right|=\frac{\left|V\right|}{\left|I\right|}$$

and

(3) ∠Z=∠V−∠I.

Therefore, by capturing the magnitude and the phase of both the voltage and current, one can determine the complex impedance with simple calculations.

Impedance spectroscopy has wide-reaching applications including many biological and biomedical sensing applications [2,3,4], photonics [5], photovoltaics [6], and mineral analysis [7]. Impedance spectroscopy is also very common for evaluating the electrical characteristics of materials, with recent studies using impedance spectroscopy to characterise naturally synthesised ZnO [8] and a ${\mathrm{HfO}}_{2}$ pH sensor [9].

Another application, though one that is not yet fully explored, is the use of impedance spectroscopy for the detection of nitrogen species in water. A recent review [10] discusses various electrochemical sensors for this purpose, but only a single impedance spectroscopy system is listed and it is for the detection of nitrites [11]. This impedance spectroscopy sensor could detect a wide concentration of nitrite with high sensitivity. One paper, not mentioned in the review, was found that measured dissolved nitrates with EIS [12].

### 1.2. Low-Cost Design

In many of these applications, low-cost design is critical to the in situ use of impedance spectroscopy as a measuring technique. For this reason, low-cost impedance devices are of interest to the general sensors community, and there was significant development in the field of low-cost impedance spectroscopy devices [13,14,15], including our prior work on a low-cost system [16,17] for use in point-of-need applications. The conference paper [16] explored an EIS analytical technique by measuring concentrations of different salts in water for a potential multiplexed detection application. All measurements were performed with a laboratory GW Instek LCR-8000G meter, but the paper mentioned that a low-cost custom hardware system is in development. A novel algorithmic back-end implementation for the low-cost device is described and tested in the journal paper [17]. On the other hand, the purpose of the current paper is the performance validation of the complete custom EIS device against an established commercial system implementation of EIS, as well as an illustrative evaluation of the custom implementation in an application-based testing environment. Instead of validating the device with artificial models assembled with passive components, as is often reported [15,18], it was decided to validate the performance of the low-cost device doing an experiment with water spiked with an analyte of interest in our ongoing water quality monitoring work [19,20,21].

### 1.3. Experimental Design

To fully evaluate the performance of the low-cost device, an experiment was designed where an analyte can be measured by the low-cost device, while also being validated against an external device without disturbing the experimental setup. Additionally, it was decided that solute concentrations must be validated with a commercially available colorimetric test so that there is semiquantitative visual evidence to support the experimental result. Typical applications for commercial colorimetric assays are in fertiliser and soil analysis, as well as for aquaculture and hydroponics. The latter tests are appropriate to water quality monitoring.

Nitrate monitoring is selected because it is specifically applicable to our efforts towards collaboration in projects related to the bioremediation of lead, where microbial growth is increased by the addition of nitrates at concentrations varying from 80–500 mg/L [22,23]. Nitrate is also of general interest in water quality monitoring because it is detrimental to the environment through the eutrophication of aquatic environments and high leakage into soil due to high-solubility in water [24], while it may also be hazardous to humans and animals by affecting the oxygen transport capacity of blood [25]. The typical nitrate concentration range is 2–20 mg/L for optimal growth of fish stock and aquatic plants [26,27]. For drinking water, the limit of nitrate concentration is defined to be 10 mg/L by the EPA [28]. The viscolor ECO colorimetric nitrate test from Macherey–Nagel GmbH & Co. KG [29] with a dynamic range of 1–120 mg/L is used, since this suits the application as the logarithmic center of the dynamic range falls on the EPA nitrate limit.

The device-agnostic experimental setup was achieved by measuring the same sample on the same electrode with a known high-performance device and the low-cost device without making any changes to the setup other than the measuring device. The high-performance device used for validation is the DropSens µStat-i 400s [30]. The experiment is to measure a set of water solutions with varying concentrations of dissolved nitrates. Additionally, the experiment allows one to gain some insight into the influence of dissolved nitrates on the impedance characteristics of water.

## 2. Materials and Methods

### 2.1. Impedance Spectroscopy Devices

**Figure 1:**
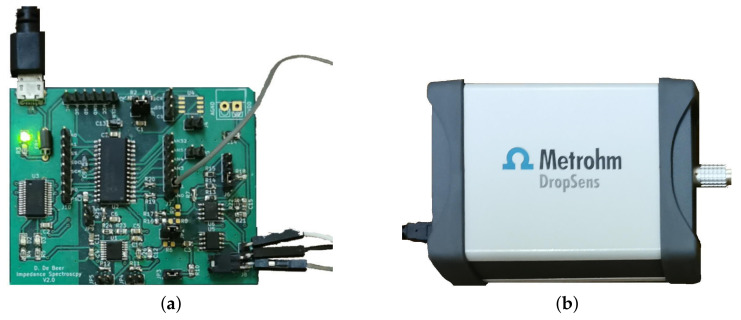
Two different impedance solutions. (**a**) Low-cost impedance analyzer (68.6 mm × 55.9 mm). (**b**) DropSens µStat-i 400s (132 × 100 mm) [30].

The two devices compared for measuring nitrate solutions are a custom low-cost impedance analyzer using an AD9835 DDS (Figure 1a), while the other is a DropSens µStat-i 400s [30] (Figure 1b), which is a single channel potentiostat that can perform the EIS experiment. Figure 1 also provides the dimensions of the two devices.

**Figure 2:**
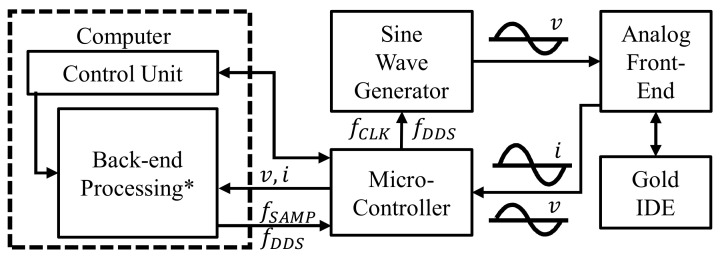
Functional diagram of the low-cost impedance analyzer device, adapted from [17]. * For more information on the back-end processing, see [17].

The basic operation of the low-cost impedance analyzer is shown in Figure 2. The system is controlled by a Python program running on a PC. This program can control various experimental parameters, such as measurement frequency, ADC sample rate, DC bias voltage, and amount of data points per sample. After the circuit acquires the voltage and current data points at a given frequency, the data are sent to the PC for postprocessing.

The internal workings of the DropSens device is proprietary information. The information that is public is that the device can be controlled by an included software suite called DropView, which allows a user to choose frequency limits, frequency points within the range, and various other experimental parameters. Among these is the voltage amplitude, which suggests that the device applies an AC voltage and measures the resulting AC current to determine impedance. Accordingly, the working operation of the two impedance analyzers are similar. The DropSens µStat-i 400s is therefore a suitable device to use as a reference to establish the performance of the low-cost impedance analyzer.

### 2.2. Nitrate Solutions

A variety of nitrate concentrations were prepared to be measured by both impedance devices after being tested with the viscolor ECO colorimetric nitrate assay. A 1000 mg/L nitrate solution was available as a high concentration source to make dilutions with distilled water. Concentrations to be measured were created by diluting the 1000 mg/L by approximate factors of 3. The factors were chosen such that some of the concentrations align with gradations of the colorimetric nitrate assay. The dilutions were made down to 1 mg/L, but this solution did not show any colour in the colorimetric test and was omitted from the experiment as its concentration could not be verified. The lowest concentration is therefore 3 mg/L. The composition of the solutions and their concentrations are shown in Table 1.

**Figure 3:**
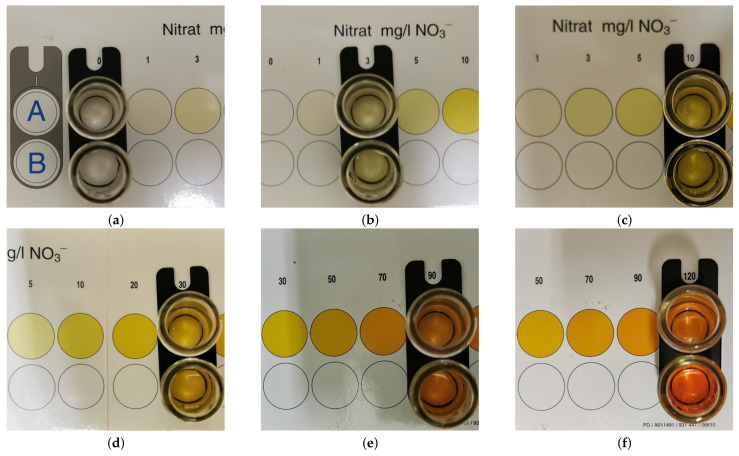
Validation of nitrate concentrations using a viscolor ECO colorimetric nitrate test. In each image, colour of bottom (coloured) test tube is matched with colour of scale underneath top (clear) test tube. (**a**) Distilled water. (**b**) 3 mg/L solution. (**c**) 10 mg/L solution. (**d**) 30 mg/L solution. (**e**) 90 mg/L solution. (**d**) 300 mg/L solution, which exceeds range of test, as seen by bottom test tube being darker than 120 mg/L gradation.

The solutions were prepared according to the proportions shown in Table 1 by using various sized pipettes to add the required amounts of 1000 mg/L solution and distilled water to test tubes. 10 mL of each of these samples were set aside to be tested with the viscolor ECO colorimetric nitrate assay. The 1000 mg/L solution was tested for completeness, but the result does not need to shown as such a high concentration is well-outside of the measurement range of the colorimetric test. Furthermore, the 1000 mg/L is not a dilution but the source, so its concentration accuracy is guaranteed by the supplier. The results of the remaining solutions can be seen in Figure 3. As seen in the figure, all solutions have approximately (within the limits of the human eye) the correct concentrations. The only measurement in the figure that does not match a gradation is the 300 mg/L solution, as it exceeds the range of the colour scale.

### 2.3. Solution Interface Setup

**Figure 4:**
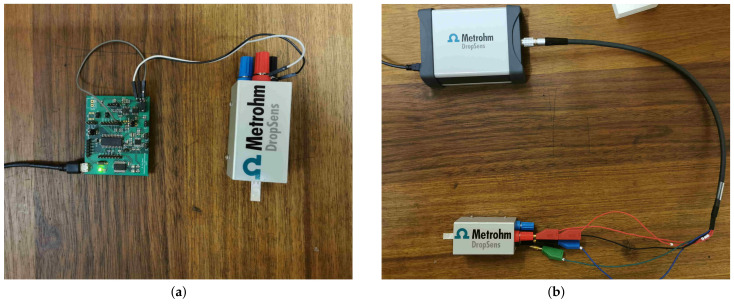
Experimental setup for two different impedance solutions. Both solutions make use of same electrode and boxed connector, which allows changing device connected to electrode without disturbing the electrode. (**a**) Low-cost impedance analyzer setup. (**b**) DropSens µStat-i 400s setup.

DropSens interdigitated gold electrodes over white plastic substrates (PW-IDEAU50) are used to interface the impedance analyzers with the solutions. Additionally, a boxed connector for screen-printed electrodes (DSC DSC4MM) is used to facilitate easy connection to the electrode, as well as easy changes to those connections. The electrode and the boxed connector can be seen in Figure 4, which shows the experimental setup for both devices.

The low-cost impedance analyzer setup in Figure 4a shows the low-cost PCB connected to a PC through USB for experimental control and data processing. The board is then connected with two wires to the working and reference ports of the boxed connector. The counter port is not connected as the gold electrode only has two connectors, which contact the working and reference pads of the boxed connector. The reference and counter ports on the PCB are shorted to set the PCB to a 2-point electrode mode. The last wire visible in the image is simply an additional measurement probe for the on-board ADC for debugging purposes.

The DropSens setup in Figure 4b shows the DropSens µStat-i 400s connected to the same PC as the low-cost impedance analyzer through a USB connection. The potentiostat is connected to the boxed connector with a proprietary cable with 5 connectors, namely: working 1, working 2, reference, counter, and ground. Ground is left unconnected as no Faraday cage is used to reduce electromagnetic interference for this experiment. Both working cables are connected to the corresponding port on the boxed connector, while the reference and counter cables are connected to the reference port. This yields an equivalent setup to the one described for the low-cost impedance analyzer.

### 2.4. Experimental Procedure

**Figure 5:**
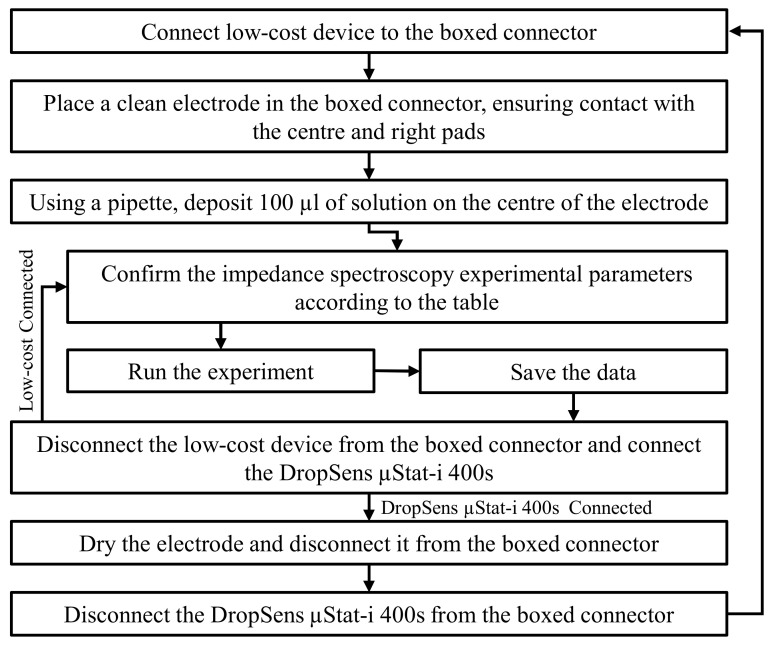
Flow diagram of experimental procedure for impedance measurement.

The experimental procedure is shown in Figure 5. Using the described procedure, all seven solutions were measured. The frequency range of the low-cost device is 60 × 10^−3^–12.5 × 10^6^ Hz, and the optimal dynamic range is 3.125–6.4 × 10^3^ Ω. The experimental parameters in Table 2 were chosen so that the magnitude of the impedance stayed mostly within the optimal range.

## 3. Results and Discussion

### 3.1. Initial Results

**Figure 6:**
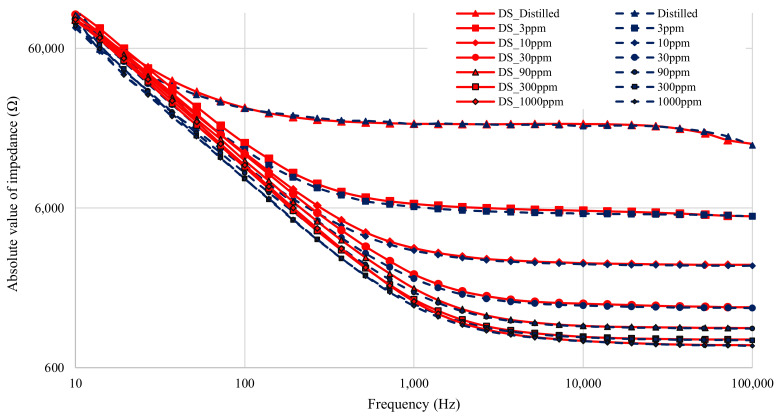
Figure showing magnitude of impedance of all seven solution measurements before calibration. All DropSens µStat-i 400s measurements are in red, with low-cost impedance measurements in blue.

**Figure 7:**
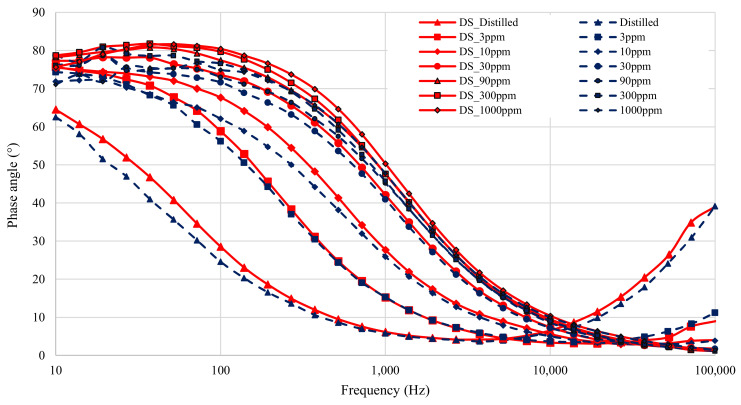
Figure showing phase angle of all seven solution measurements before calibration. All DropSens µStat-i 400s measurements are in red, with low-cost impedance measurements in blue.

The results from all seven solutions are shown in Figure 6 (magnitude) and Figure 7 (phase). The results span four decades of frequency, from 10–100 × 10^3^ Hz. The total dynamic range of the test impedance is 40. The phases measured range from 0–82${}^{\circ }$. The nitrate measurement experiment therefore tested the limits of the low-cost impedance analyzer, as both the magnitude and phase measurements spanned a large portion of the device’s range. In fact, the test exceeded some limits, since the maximum measured impedance exceeded the rated impedance for the low-cost device, which is 6.4 kΩ.

**Figure 8:**
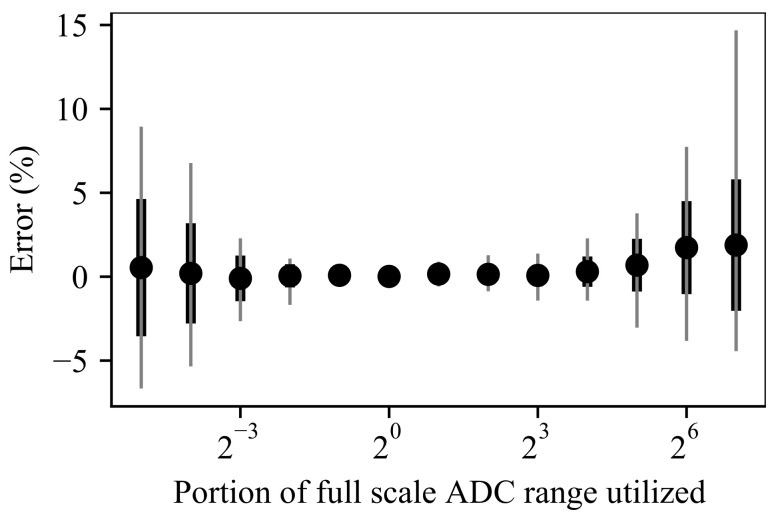
Measurement accuracy for saturated and nonsaturated measurements. Graph shows average, extremes, and standard deviation when measuring above and below the full ADC range with constant noise. (Reproduced from [17] with permission from IEEE).

As seen in Figure 8, the accuracy of the device decreases when the size of the signal waveforms being measured deviate from the full ADC range. The reasons for this are discussed in [17], but for the purposes of this paper one simply needs to note that the accuracy of the device is within 5% in the ADC range of $\frac{1}{16}$ to 64. This corresponds to an impedance range of 3.125–6.4 × 10^3^ Ω, which is the rated dynamic range of the device. Values above 6.4 kΩ can still be measured, but the accuracy will decrease according to Figure 8.

Figure 6 shows that the magnitude results from the low-cost analyzer closely matches that of the DropSens µStat-i 400s at high frequencies (low impedances). A slight difference that occurs, however, is an offset issue where the low-cost analyzer reports values that are lower than that of the DropSens µStat-i 400s. This offset issue is resolved by correctly calibrating the low-cost device for its transimpedance amplifier gain. At low-frequencies, the low-cost analyzer reports significantly lower impedance values. As the double-layer capacitance is dominant at lower frequencies, it can be considered the primary source of the offset error. The error is likely due to differences in the measured double-layer capacitance caused by differences in the applied voltages between the two devices [31,32]. The measured applied voltage on the low-cost device is 11.15% higher than the specified amplitude of 100 mV from Table 2. As the double-layer capacitance is proportional to the applied voltage, it is expected that the 11.15% difference in applied voltage would lead to the double-layer capacitance measured by the low-cost device to be 11.15% higher than the double-layer capacitance measured by the DropSens device.

Figure 7 shows the measured phase for the different nitrate solutions. The graph shows that the phase accuracy of the low-cost device is compromised when the impedance magnitude is high. The impedance is usually high at low frequencies in this experiment, so the phase measurements at low frequencies are noisy. This is caused by the current signal being sampled by the low-cost device becoming very small at high measured impedance. When the current signal is so small the zero-crossing becomes harder to detect and phase angle measurements are less accurate. Another point to note is that the phase offset between the devices increases at low frequencies. This difference could once again be attributed to differences in the double-layer capacitance.

The results shown in Figure 6 and Figure 7 are before any calibration was done. The gain of the low-cost impedance analyzer is dependent on a feedback resistor and therefore needs to be calibrated to accommodate for the ±5% tolerance on the resistor. Data correction also needs to be applied to compensate for the variation in double-layer capacitance as the two impedance analyzers did not have identical sinusoid voltages. The gain calibration only effects the magnitude measurement, while the double-layer capacitance compensation affects both the magnitude and the phase.

### 3.2. Calibration

The gain calibration of the low-cost device can be achieved in one of two ways. Either an impedance can be measured with another device and the gain can be set according to the differences in the reported results between the two devices, or one can measure a stable known impedance and calibrate the device to correctly report the value of the known impedance. For typical use in the field, the second approach would be used. But since another device was used for validation, the first approach will be used for calibration.

The first stage of the calibration is very simple. The transimpedance amplifier gain has an equal multiplicative affect on all impedance magnitudes and can therefore be retroactively calibrated by multiplying all magnitudes by a calibration factor. This calibration factor, *K*, can be achieved at a single point by comparing the expected, *E*, and measured, *M*, values:

(4) $$K=\frac{E}{M}$$

This can be done at multiple points, and an average calibration factor can be chosen to apply to the entire set. This value was found to be K=1.013, which suggests that the value of the transimpedance amplifier gain is 1.3% off from its theoretical value.

The compensation of the double-layer capacitance is more complicated, but can still be computed in a simple spreadsheet. One first needs to convert the magnitude and phase data into resistance and reactance, and then one needs to apply a frequency-dependent offset to the reactance to compensate for the differences in capacitance. Once the offset has been applied, the values can be converted back into the magnitude and phase representation. The effective series capacitance is reduced from 100 nF before calibration to 91 nF after calibration. Both devices now measure 91 nF as the series capacitance, which must be increased by 9.9% to equal the precalibration value of 100 nF. The increase is in good agreement with the 11.15% difference in applied voltage between the two systems.

**Figure 9:**
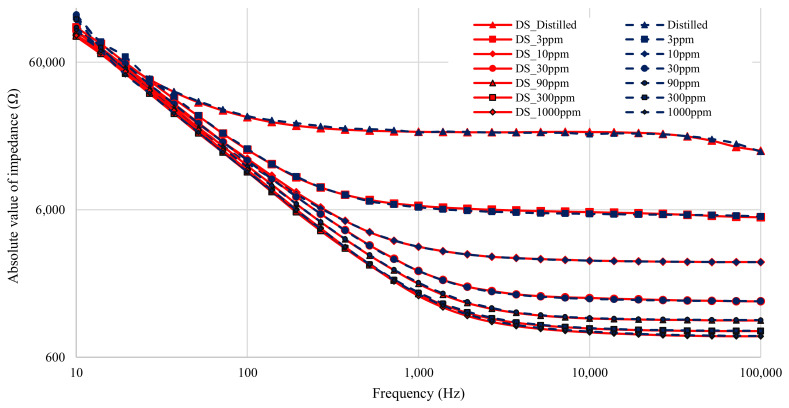
Figure showing magnitude of impedance of all seven solution measurements after calibration. All DropSens µStat-i 400s measurements are in red, with low-cost impedance measurements in blue.

**Figure 10:**
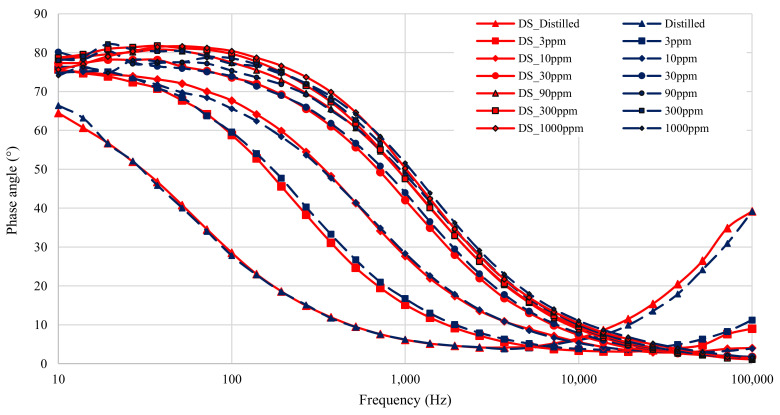
Figure showing phase angle of all seven solution measurements after calibration. All DropSens µStat-i 400s measurements are in red, with low-cost impedance measurements in blue.

The results from all seven solutions after the calibration was performed are shown in Figure 9 (magnitude) and Figure 10 (phase).

As seen in Figure 9 the magnitude results between the two devices correlate very closely once the low-cost device is properly calibrated to match the DropSens µStat-i 400s. The average absolute error across the entire range is 2.47%, but the error is reduced to 1.28% when only considering impedance measurements within the rated range of the low-cost device.

The phase measurements, which can be seen in Figure 10, also show a significant improvement after calibration. The typical phase error is 1.24° across the full range with a phase error of 0.96${}^{\circ }$ within the rated impedance range of the low-cost device. All error values are calculated by summing the absolute values of the measurement errors and dividing by the number of data points.

### 3.3. Nitrate Analysis

The concentration of nitrates in solution has a large impact on both the magnitude and the phase measured by the device. As the quantity of nitrates in solution increases, the magnitude of the impedance becomes smaller. An important feature of the phase graph is the peak phase tending to approximately 75${}^{\circ }$ as the concentration decreases. This is similar to results found by Alahi et al. [12], where 110 Hz is the point at which the reactance change as a function of concentration is at a maximum. In Figure 10, the peak phase corresponds to the point of maximum reactance which would correspond to the reactance point that is most sensitive to changes in nitrate concentration. Another feature of the phase graph is the increase in phase that can be seen at high frequencies for the measurements with high impedance, such as the distilled water. The reason for this is largely unknown. A possible hypothesis is that a parallel parasitic element exists that can only affect the measured magnitude and phase if the impedance is sufficiently high. Further investigation into this phenomenon is required, and is planned as part of future work.

**Figure 11:**
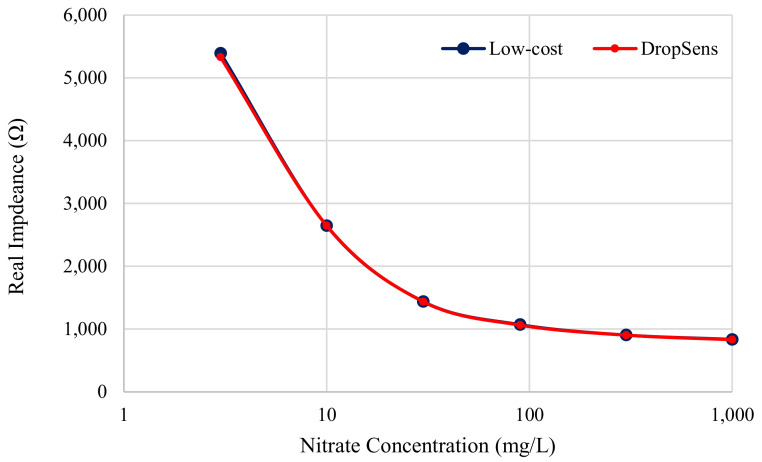
Functional diagram of low-cost impedance analyzer device.

Figure 11 shows the real impedance as a function of nitrate concentration. The relationship between these quantities, however, is not linear. The impedance seems to be reaching a lower limit as the concentration of nitrates increases, which suggests that the data follow a sigmoidal curve. Sigmoids are a common feature in concentration calibration curves, although typically the sensor would be designed to operate within the linear region of the sigmoid. Alahi et al. [12] measured the impedance for a concentration range of 1–10 mg/L and found the region to be linear. The region for concentrations below 10 mg/L in Figure 11 is linear, and therefore matches these findings.

In terms of the sensor performance, the results show that the limit of detection (LOD) for nitrates with this method seems to fall below the range of concentrations measured in this experiment. Alahi et al. found their LOD to be 1.06 mg/L, and if one can extrapolate that this system may achieve a similar LOD, it shows great promise for using impedance spectroscopy for this application.

### 3.4. Performance Comparison

Overall, the low-cost impedance analyzer performed as expected when compared to that of a higher end device such as the DropSens µStat-i 400s. Table 3 summarises the performance of the devices.

### 3.5. Cost Analysis and Comparison

A cost analysis and comparison is included to justify the classification of the device as low-cost. For convenience, all prices in this document are given in USD. When converting from South African Rand (ZAR), a conversion rate of 14.38 ZAR/USD (1 September 2021) is used. As seen in Table 4, the total unit cost of the low-cost device is approximately $52.4. This cost is based on the original purchase prices in South Africa in Q3 of 2019. Shipping and import duties have a significant impact on component prices in South Africa. The price of the AD9835 chip from Analog Devices, for example, costs only $9.18 when purchased in the United States, while the price is $46.07 in South Africa as of Q3 2021. It follows that the cost of this device would be considerably lower if it were produced in a country with cheaper access to components—independent of costs due to economies of production scale. For this reason, the table also includes the total unit cost if the device were to be produced in the USA in 2021. This cost disparity is one of the driving factors for the design and production of low-cost devices in South Africa (and in other similar economies).

The cost of the DropSens µStat-i 400s was $6,910 when purchased in Q4 2020. This price is orders of magnitude higher than that of our low-cost device, but it is not a fair comparison as the commercial device has multiple additional features, and the cost needs to cover significant engineering costs as well as numerous overheads.

In Table 5, the cost and performance of the device presented here is compared to that of other published works. From the table, one can see that this device performs favourably in terms of the magnitude and phase accuracy as well as the frequency bandwidth. The accuracy of the device is noteworthy as the ADC is only 10 bit while the most accurate device by Munjal et al. [15] uses a 12 bit ADC. The cost is also competitive when the device cost outside of South Africa is considered. This cost comparison raises an important point: it is complex to compare the cost of different devices from different regions and different times. The reporting of cost can also differ from paper to paper due to certain costs being included or excluded by different authors. The difference between component prices in small and large quantities can also be considerable. With all these discrepancies in mind, one needs to be aware that the cost comparison is very approximate and there are large uncertainties attached to the presented values.

## 4. Conclusions

Measuring a range of dissolved nitrates proved to be a rigorous test for the low-cost impedance analyzer. The test covered most of its frequency range and dynamic range in a setting that is very similar to its intended application. The low-cost device measured magnitudes with an average error of 1.28% and measured phases with an average error of 0.96${}^{\circ }$. These results are a promising start for this low-cost impedance analyzer prototype.

Future work for the nitrate quantification project includes a more comprehensive analysis of the impact of dissolved nitrates on impedance measurements. This would include performing experiments down to lower nitrate concentrations so that a limit of detection can be calculated for the device and measuring nitrates in complex matrices as opposed to distilled water. Another avenue for future work is to iterate on the design of the low-cost impedance analyzer to reduce its cost or increase its performance without incurring additional cost.

## Figures and Tables

**Table 1:** 1000 mg/L nitrate dilutions to achieve various concentrations of nitrates in 50 mL of distilled water.

	1	2	3	4	5	6	7
Desired Concentration (mg/L)	0	3	10	30	90	300	1000
Proportion of 1000 mg/L (%)	0	0.3	1	3	9	30	100
Volume of 1000 mg/L (mL)	0	0.15	0.5	1.5	4.5	15	50
Volume of distilled water (mL)	50	49.85	49.5	48.5	45.5	35	0
Label in Figure 3	(**a**)	(**b**)	(**c**)	(**d**)	(**e**)	(**f**)	N/A

**Table 2:** Experimental parameters for impedance spectroscopy measurements.

Parameter	Value
Frequency Start	10 Hz
Frequency Stop	100 kHz
Points	29
Voltage Amplitude	100 mV
Settling Time	1 s

**Table 3:** Comparison of some impedance spectroscopy performance metrics.

	Our Impedance analyzer	DropSens µStat-i 400s
Frequency Range	60 × 10^−3^–12.5 × 10^6^ Hz	1 × 10^−3^–1 × 10^6^ Hz
Frequency Resolution	11.64 mHz	1 mHz
Impedance Range	3.125–6.4 × 10^3^ Ω (1 range)	100–10 × 10^6^ Ω (9 ranges)
Impedance Accuracy	±1.28%	±0.6%
Measurement Duration	3 min	12 min

**Table 4:** Cost analysis of impedance spectroscopy prototype.

Component	Original Cost ($)	2021 Cost ($)
PCB	15.3	4.50
AD9835	25.9	9.18
FT232RL	5.2	4.25
CLC1005	3.6	3.41
Passives	2.6	1.50
Total	52.4	22.84

Costs were converted to USD using a rate of 14.38 ZAR/USD. Original prices given are from Q3 2019 in South Africa.

**Table 5:** Comparison between this work and selected impedance measurement systems.

Reference	Frequency Bandwidth	Max Sampling Frequency and Resolution of ADC	Measurement Deviation	Cost
Breniuc et al. [33] (Romania, 2014)	1 × 10^3^–100 × 10^3^ Hz	1 MHz, 12 Bit	Relative Error of the Impedance Modulus Measurement is in the range of ±2%	Approx. 36$
do Amaral et al. [34] (Brazil, 2011)	50–1 × 10^6^ Hz	12.5 kHz, N/A	Magnitude Mean Deviation of 2.9% and Phase Mean Deviation of 0.69${}^{\circ }$	Approx. 48$
Michalikova et al. [4] (Czech Republic, 2014)	1 × 10^3^–1 × 10^6^ Hz	N/A	Impedance Relative Deviation of −4.52% to 5.98% with Mean Value of −0.02%	Approx. 24$
Munjal et al. [15] (Germany, 2020)	DC to 10 MHz	1 MHz, 12 Bit	Impedance Magnitude Standard Deviation of 0.5% and Impedance Phase Standard Deviation of 0.55${}^{\circ }$	Approx. 24$
This Paper (South Africa, 2021)	60 × 10^−3^–12.5 × 10^6^ Hz	1.2 MHz, 10 Bit	Magnitude Absolute Mean Deviation of 1.28% and Phase Absolute Mean Deviation of 0.96${}^{\circ }$	Approx. 52$ (22.84$ based on US pricing)

## Data Availability

The data presented in this study are openly available in Mendeley Data at doi: 10.17632/8cp6n8sm4p.1.
